# Elevating Local Voices: Evaluation of California’s Local Aging and Disability Action Planning Grant Program

**DOI:** 10.1093/geroni/igaf122.1109

**Published:** 2025-12-31

**Authors:** Elizabeth Bogumil, Pauline Martinez

**Affiliations:** University of California, Riverside, Riverside, California, United States; Betty Irene Moore School of Nursing, University of California, Davis, Davis, California, United States

## Abstract

The University of California, Davis, Family Caregiving Institute evaluated the California Department of Aging’s Local Aging and Disability Action Planning (LADAP) grant program, a $4.5 million initiative funding 21 grantees—city and county governments, nonprofits, and consortia—to develop inclusive, community-driven aging and disability action plans. Aligned with California’s Master Plan for Aging (MPA), LADAP prioritizes historically underserved communities. The mixed-methods evaluation, including surveys, document analysis, and interviews, centered community voices, ensuring their perspectives shaped local planning and state policy. Processes like iterative feedback, monthly learning labs, and office hours strengthened grantees’ capacity to engage communities in meaningful ways, offering a replicable model for community-engaged scholarship. Grantees tailored strategies to local contexts, engaging cross-sector leaders and fostering partnerships with elected officials, government agencies, Area Agencies on Aging, and community-based organizations. These collaborations expanded outreach and inclusion of diverse demographic and geographic communities. Findings demonstrate how local planning informs state policy and underscore the need for sustained local-state coordination. Key strategies—leveraging trusted community leaders and fostering cross-sector partnerships—enhanced LADAP’s reach and impact, ensuring policies are responsive to community needs. These insights have informed program adjustments, advancing health, well-being, and equity objectives within engaged communities. As MPAs gain traction nationwide, LADAP highlights the value of local action plans in driving equitable aging policies. The evaluation’s findings and processes provide a scalable model for other states, emphasizing community-centered engagement and iterative learning. This approach strengthens state efforts, ensuring policies are inclusive, sustainable, and rooted in community-driven insights.

